# Supplementary material for: Patterns of Intron Gain and Loss in Fungi
Source: PLoS Biol. 2004 Nov 30;2(12):e422. doi: 10.1371/journal.pbio.0020422 (PMC532390; doi:10.1371/journal.pbio.0020422)
Supplement: Table S1 — Also available at http://genes.mit.edu/NielsenEtAl/. (4.3 MB ZIP). [file pbio.0020422.st001.zip › NielsenEtAl/html/275.html]

AN0129.1.NCU03426.1.MG03130.1.FG04296.1


```
 CLUSTAL W (1.82) Multiple Sequence Alignments - Introns Inserted


Sequence 1: NCU03426.1	712 aa
Sequence 2: FG04296.1	698 aa
Sequence 3: MG03130.1	699 aa
Sequence 4: AN0129.1	689 aa
Alignment Length: 768 aa
Number Identitical Residues: 277 aa
Alignment Score (without introns) 14315


MG03130.1 	MATIALPRPIPAHRASSGLS-----LSSSSIAIDSINANPVPNKHLPVCPPGPVPRSAPH
NCU03426.1	MATIALPPPFDNYRSTSTIG---SMTTTITIEQPPSHPTPVPNKHIPVCPPGTASRDDAD
FG04296.1 	MTTVTLARPIPPHRPSSTITPPLSLESSTTSSSSP-CSGPIPNKHLPICPTGPACPDELD
AN0129.1  	MATVVVQQPLRHTTPPPGTV-----APALTLNRTS---SPVPNKHIPVCPTGPSPVSSRT
          	*:*:.:  *:    ...         .: :    .    *:****:*:**.*.   .   

MG03130.1 	PHDNHPPTLETLDSDHNSVNESSQSILFPPDAFPRFSSADGGSLIVYQLTPRDIADALDH
NCU03426.1	TPPPSPWPVTNGADEHS----PPRSFLYPPHRFHHVDS---GHLSLYEIDAADVAAALDH
FG04296.1 	TPPPSPP-----AQDQL----QQTSALYPLDSFKRVDS---GELSIYELDAFNVAEAVDV
AN0129.1  	SSPAPK-------DDVVL---PTSSPLHPPDAFPKISE----SPLLYSIEIEILVAAVDH
          	.            .:         * *.* . * :...       :*.:    :. *:* 

MG03130.1 	IARQPLPDPSAVFPWLHGLHPQNHIQQAFFVQRR--RSLRRTPHCLRSVTLVKADGDLST
NCU03426.1	LSRQPLPDPAHVFPWYHGLHPRNQVQQHFFTSPRTWRAQKKTPSCLRGITLVKADGDLNF
FG04296.1 	ASRQPLPDPALVFPWFHGLHPQNHVQQSFFAARR--RATRPTPSCIRALTLVKADGDLNY
AN0129.1  	CSSQPLPDPNLMFPWLHGLHPENHLQVGFFTNRR--RSLRRTPKCWRGLTIVKVGGDLST
          	 : ******  :*** *****.*::*  **.  *  *: : ** * *.:*:**..***. 

MG03130.1 	SRLKGAIAPQEFLQSDT-AEFIDVDPKDGFSVRNFQIQSAKSAMTSDIVVYGDD---EPL
NCU03426.1	SRLKGAIAPYEFLHSSS-AEFIDVDPEHGFSVRNFQIQAAKAAMTSDIIVYGDN---EAA
FG04296.1 	ARLKGAIAPNEIIQPNSPARFIEADPREGFSVRNFQIQTVKTAVLSDIIVYGQD---LNK
AN0129.1  	SRLKGAITPDAILAPSG-LEFLATDPREGFSVRNFQIQTAKVAPLSDIVVYGEDGVTAKQ
          	:******:*  :: ..   .*: .**..**********:.* *  ***:***::. :   

MG03130.1 	VRKVGWDIANAQQAWRQKHIDQGDLALPEYNTFVCLS~RFSSFEKNHPEIVAVDSGGRLT
NCU03426.1	VRKLGWDIASAQHKWRYKHELQC-HWVPEYNTFICLS~PFDEFEQNHREIVAVDSTGNLT
FG04296.1 	TRKVAWDIANAQKLWREKHQDQG-EWMPEYHTFVCTN~PFSKFEENHSEIVAVDESGHLT
AN0129.1  	LLEVASRIATAQHHWRLKHDHEQ--TLPSYNTFVVSC1PFSDIERRAPNLVAVNSSGQLT
          	  ::.  **.**: ** **  :    :*.*:**:     *..:*..  ::***:. *.**

MG03130.1 	GRVVDLFSQ----------------ERREMYAMTKASEIAPNVFLGPTPD-------PST
NCU03426.1	GNVLDFVNQ----------------ERLEMYLMTQASEIAPNVWMGPTPD-------PII
FG04296.1 	GNVLDFFSQ----------------ERREMWAMTEASEFSKNVFMGPTPE-------QGS
AN0129.1  	NQVVDLCESTECCARIRSNKRLVQLERLEMCTMSRATEFAHNVWQGPTPDYLLQAGASGT
          	..*:*: ..:...:   :..   . ** **  *:.*:*:: **: ****:   .:.:   

MG03130.1 	GEDQDYDIFIECNDMAQLSPVCLQAIVGIPDEVI---QQVFLEFPSSGSILPPTWSQAEA
NCU03426.1	EEDNGYHIQLECSDMGRANTCAAFLQALAEDGLGDVPEPVHFDFPSSGSFLVPDYSHEDA
FG04296.1 	PEEQKYDILIECSDLGRLNPLALQLIAESPDQDG---KQRFLDFPSSGSILPPTWSHSEA
AN0129.1  	PSADAFDLMIDANDLAGIP--GPRYLANLAKRLAK--EPQRMEFPSSGSLLTPSGESRDL
          	 . : :.: ::..*:.          .   .   .  :   ::******:* *  .  : 

MG03130.1 	DGILDTCKWLYHLAQGTCPDPVD------IDNGEAMTGAGSQS---KFPPRKILIHCQDG
NCU03426.1	DALLDTCKWLWHLSHGTRPEKSS-------DTGRDKSWP-------PLSPRKILIHCADG
FG04296.1 	DGVLETCKWIYHLAHGSYPEPDPGLSIGDVDAEGDSPMPSDHGSAFEVRPHKILLHCADG
AN0129.1  	DDLINTLRWIYYLAN---PEVVET------DAEGDVSMDIP-----PRKTRRILIHCPDG
          	* :::* :*:::*::   *:          *     .            .::**:** **

MG03130.1 	YTESTMLAIAYVSYATGMPVPEAWLHLHTVKKRNFFAYPTDVALLTAIAPRLLSASPVHA
NCU03426.1	YTESTVLAIAYYSYSTGLPIPEAWLKLHTIMKRNFFAYPSDVNFLATIAPRLLQESPMGT
FG04296.1 	YTESTMLSIAYFSYSTGRPVPEAWLQLHTEKKRNFFAYPTDVALLTSITPRLLRESPVCK
AN0129.1  	YTESSLLVIAYTMFAEGISASEAWLRLHREKKRNFFAYPSDVAYLKNVQERILQESPANP
          	****::* ***  :: * . .****:**   ********:**  *  :  *:*  **   

MG03130.1 	ETASLAEMTAMLKSERNEPKWLAGLDGSLPSRILHYMYLGNLGHANNPDLLRELGIGQIL
NCU03426.1	D-RSLAQVTELVR---NEPKWWTGFDGSLPSRILDYMYLGNLTHANNPDLLRAMGIGQIL
FG04296.1 	G-LSLTDITHLTR---DEPSWFAALDGSFPSRVLDYMYLGNLGHANNPDLLRALGITQIL
AN0129.1  	P-------TYRVS----DPQWFKYCDGSLPSRILPYMYLGNLSHANNPEMLWELGIRRVL
          	        *        :*.*    ***:***:* ******* *****::*  :** ::*

MG03130.1 	SVGETAMWRDGELEQWGPENVCIVENVQDNGIDPLTCEFQRCLEFIE1RGRRNGTATLVH
NCU03426.1	SVGEMAMWRDGELQEWGEENTCIVRRVQDNGIDPLTDEFERCLEFID1RGRRLGTATLVH
FG04296.1 	SVGEIAMWREGDLEAWGKENVCVVQGVQDNGIDPLTEEFARCLDFID1RGRRNGTATLVH
AN0129.1  	SIGEAVTWSDSDIARMGAENLMHITKVQDNGIDPLTQEFDRCLEFIR1KGKSDGAATLVH
          	*:** . * :.::   * **   :  ********** ** ***:**  :*:  *:*****

MG03130.1 	CRVGVSRSATICIAEVMKELKLSVPRAY~SFVRARRLNVIIQPHLRFA~YELLKWEELLQ
NCU03426.1	CRVGVSRSATICIAEVMRSMGMSFPRAY~CFVRARRLNVIIQPHLRFA~YELLKWEEFLR
FG04296.1 	CRVGVSRSATICIAEVMRSKGMSFPHAY~CYVRARRLNVIIQPHLRFA~YELLKYEEALQ
AN0129.1  	CRVGVSRSATICIAEVMASLNLSFPRAY2CYVRARRLNVIIQPHLRFV2YELLKWEELQQ
          	***************** .  :*.*:** .:****************. *****:**  :

MG03130.1 	LH------------WNGK-----------IQRELEWSEITREIALMNRPYAR
NCU03426.1	SNQETKDHGNYTLTADGKDDNESSSEGQGIMRELEWGEIAREVALMNKPYSR
FG04296.1 	Q--------------DGDPDAE-------VKRTLEWAEIAREIALMNRPYSR
AN0129.1  	KK-------------NKQP----------VKRELEWATVAREIALMNKPYSR
          	               : .           : * ***. ::**:****:**:*
```
